# Tetralol derivative NNC-55-0396 targets hypoxic cells in the glioblastoma microenvironment: an organ-on-chip approach

**DOI:** 10.1038/s41419-024-06492-1

**Published:** 2024-02-10

**Authors:** Clara Bayona, Lía Alza, Teodora Ranđelović, Marta C. Sallán, Anna Visa, Carles Cantí, Ignacio Ochoa, Sara Oliván, Judit Herreros

**Affiliations:** 1 Tissue Microenvironment (TME) Lab, Institute for Health Research Aragón (IIS Aragón), Aragón Institute of Engineering Research (I3A), University of Zaragoza, 50018 Zaragoza, Spain; 2 Calcium Cell Signaling, IRBLleida, University of Lleida, Rovira Roure 80, 25198 Lleida, Spain; 3 Centro de Investigación Biomédica en Red de Bioingeniería, Biomateriales y Nanomedicina (CIBER-BBN), Instituto de Salud Carlos III, 50018 Zaragoza, Spain; 4 Present Address: Centre for Haemato-Oncology, Barts Cancer Institute, Queen Mary University of London, Charterhouse Square, London, EC1M 6BQ UK

**Keywords:** Cancer microenvironment, Cancer models, Microfluidics

## Abstract

Glioblastoma (GBM) is a highly malignant brain tumour characterised by limited treatment options and poor prognosis. The tumour microenvironment, particularly the central hypoxic region of the tumour, is known to play a pivotal role in GBM progression. Cells within this region adapt to hypoxia by stabilising transcription factor HIF1-α, which promotes cell proliferation, dedifferentiation and chemoresistance. In this study we sought to examine the effects of NNC-55-0396, a tetralol compound which overactivates the unfolded protein response inducing apoptosis, using the organ-on-chip technology. We identified an increased sensitivity of the hypoxic core of the chip to NNC, which correlates with decreasing levels of HIF1-α in vitro. Moreover, NNC blocks the macroautophagic process that is unleashed by hypoxia as revealed by increased levels of autophagosomal constituent LC3-II and autophagy chaperone p62/SQSTM1. The specific effects of NNC in the hypoxic microenvironment unveil additional anti-cancer abilities of this compound and further support investigations on its use in combined therapies against GBM.

## Introduction

Glioblastoma, the most common malignant brain tumour in adults, is characterised by rapid growth and bad prognosis. Despite current treatment, consisting of a combination of surgery (when possible), radiotherapy and chemotherapy using Temozolomide [1], tumour recurrence is inevitable. At present, GBM remains an incurable disease for which new therapeutic strategies are needed.

In the last years, the key role of the microenvironment in tumour progression has been elucidated. Different microenvironments co-exist in GBM, with populations of glioma stem cells adapting to the distinct conditions, thus contributing to GBM cell heterogeneity and plasticity [2]. The central hypoxic/peri-necrotic niche drives adaptation to low oxygen partial pressure of tumoral tissues, which promotes invasion and drug resistance, significantly worsening the prognosis of patients [3, 4]. Hypoxia triggers a complex signalling network that induces consistent changes in gene expression, contributing to adaptative responses such as autophagy that result in a more aggressive phenotype and reduced sensitivity to therapy [5–7]. Hypoxia-induced signalling emanates from the activation of the hypoxia-inducible factor 1α (HIF-1α). HIF-1α is an oxygen-sensitive transcription factor whose stability is reduced in the presence of oxygen because of post-translational modifications. In normoxic conditions, prolyl hydroxylases (PH) hydroxylate proline residues of HIF-1α, resulting in its ubiquitination and its subsequent proteasomal degradation [5, 8]. However, hypoxic conditions inhibit PH activity thus avoiding HIF-1α degradation. As a result, HIF-1α is translocated to the nucleus where it binds HIF-1β to transcribe a myriad of genes involved in adaptation to hypoxia and cancer progression [5, 9]. The dependency of cancer cells on HIF factors when growing in hypoxia points to HIF-1α as a candidate for pharmacological intervention [10].

The administration of tetralol derivatives, mibefradil and NNC-55-0396 (NNC), induces apoptosis in a variety of cancer cells cultured in vitro [11]. In addition, both compounds have been shown to delay tumour growth in GBM xenograft models [10, 12, 13]. Furthermore, mibefradil was tested in combination with TMZ in clinical trials, showing good tolerance and positive responses in GBM patients [14]. The cytotoxicity of these compounds has been attributed to the blockade of T-type calcium channels (TTCCs), which participate in activating pro-proliferative and pro-survival pathways in cancer cells [15]. However, we have reported recently that, in fact, induction of GBM cell apoptosis by NNC is mainly due to calcium mobilisation and activation of ER stress cascade [11, 16]. Mibefradil was also shown to deregulate basal autophagy of melanoma cell lines [17], a cell process further enhanced by chemotherapy. Therefore, targeting autophagy has been proposed to sensitise cells to apoptotic stimuli. Moreover, the co-administration of TMZ and the lysosome inhibitor chloroquine has been explored in clinical trials for GBM [18, 19].

However, there is no information about the effect of NNC on the different regions of the microenvironment present in heterogeneous tumours such as GBM, partly due to the limitations of conventional in vitro models recapitulating the intricate and dynamic in vivo landscape of the tumour microenvironment [20]. Recreating this complexity has been a challenge, especially considering the gradients of hypoxia and normoxia within the tumour mass, which induce the switch to different cell phenotypes in each region [21, 22]. The current scenario of cancer therapy is fraught with obstacles, as evidenced by the low success rate of anti-cancer drugs in clinical trials. Specifically, less than 10% of such drugs are ultimately commercialised, with a substantial proportion failing during the final stages of clinical development despite obtaining promising results in preclinical models [23]. The efficacy of anti-tumoral treatments is not homogeneous throughout the tumour, and it is therefore essential to study their effects on the tumour mass, including all its niches. Moreover, the use of in vivo models to investigate human outcomes presents a challenge as the findings may not always be generalizable across species. This has led to medications and vaccinations that have progressed through preclinical development. However, some have failed to show effectiveness or caused life-threatening toxicity, resulting in human trials being discontinued. It is also concerning that certain drugs could be efficient for humans, and yet have not been included in clinical trials because of inconclusive results in animal models [24–26].

Three-dimensional microfluidic models, based on the organ-on-chip concept, emerged as a solution to address these limitations. Organ-on-chip models better mimic the tumour microenvironment, mechanical properties and physico-chemical characteristics of solid tumours, while allowing a continuous flow and controlled supply of nutrients or drugs [27, 28]. Microfluidic models have been able to simulate the tumour microenvironment [21, 29], tumour invasion and metastasis [22, 30–32], vascularisation [33–37], extravasation [34, 38], tumour-immune system interaction [39–41] and are suitable for drug screening [42, 43], offering systems that come increasingly close to the in vivo conditions of different tumours, including GBM [44–46].

**Fig. 1:**
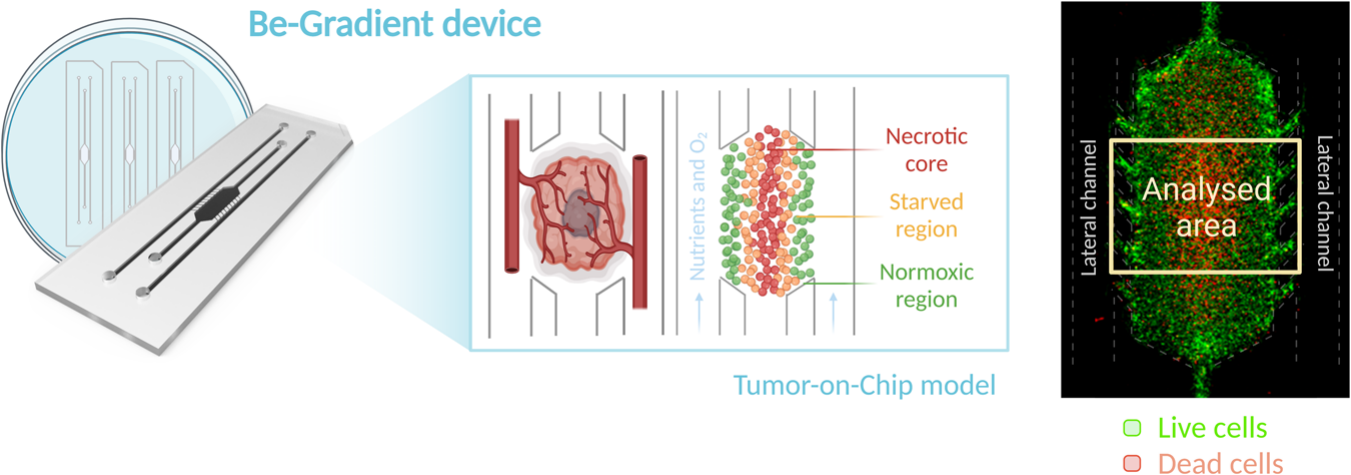
GBM-on-a-Chip model based on a microfluidic device. The image on the right shows a microdevice as seen through the fluorescence microscope [2x]. Live cells are marked in green and dead cells in red. The dashed yellow square exemplifies the analysis area chosen for processing the images obtained. The dimensions of the device are 4 × 2 mm central chamber, 0.7 mm lateral channel width, 250 µm height.

Using the organ-on-chip technology, we have been able to establish a microfluidic model that allows the simulation of a solid tumour in vitro with a hypoxic region in the centre of the tumour mass [21], due to the use of biocompatible, gas-impermeable materials (Fig. 1). This system recreates the different spatial regions within a tumour: the central necrotic core, the starved middle zone and the normoxic area. The outermost region of the tumour is supplied with nutrients and oxygen through the two side channels of the device, creating diffusion gradients similar to those occurring in the tumour in situ. In addition, the side channels allow the application of anti-tumour compounds that enable real-time monitoring of the effects of the drug on tumour viability parameters.

In this study, we aimed to evaluate the potential effects of NNC on the GBM microenvironment. The use of an organ-on-chip model (GBM on a chip) that simulates the tumour landscape in vivo has allowed us to observe how NNC acts in the different tumour zones and to show its preferential effect in hypoxic regions.

## Materials and methods

### Reagents and antibodies

Glioblastoma cell lines, U-251 MG and A-172, were obtained from the American Tissue Culture Collection (ATCC). All cell culture reagents were obtained from Thermo Fisher Scientific, Massachusetts, USA. Reagents were obtained from the following companies: NNC 55-0396 (357400-13-6) from Alomone, Israel; and bafilomycin-A (sc-201550) from Santa Cruz Biotechnology, USA. Primary antibodies used were β-actin (Merck Sigma-Aldrich, USA; A5441), P62/SQSTM1 (Novus Biologicals, Spain; NBP1-48320), LC3A (Novus Biologicals, Spain; NB100-2331), HIF-1α (BD Biosciences, New Jersey, USA; 610958) and Ki-67 (Santa Cruz, sc-23900). Secondary antibodies used were Alexa Fluor 488 goat anti-rabbit (Invitrogen, Massachusetts, USA; A11008) and goat anti-mouse FITC (Fisher AP130FMI).

### Cell culture

Cells were maintained in Minimal Essential Medium (21090-022) with 10% of inactivated foetal bovine serum (FBS) (10270098), 20 units/mL penicillin and 20 μg/mL streptomycin (15070-063), L-glutamine 2 mM (25030-024) and 1% non-essential amino acids (11140-035). U-251 MG was stably transfected with green fluorescent protein (GFP) following the protocol already described [47].

A-172 and U-251 MG cell lines were authenticated by short tandem repeat profiling (StabVida, Portugal) following purification of genomic DNA. Cell lines were passaged for 20-25 passages. Mycoplasma was tested by PCR to guarantee mycoplasma-free cells. Mycoplasma positive cells were discarded or treated with Plasmocin (InvivoGen, California, USA; ant-mpt-1).

### 3D cell culture in microfluidic devices

Microfluidic devices were used following the protocol previously described [21]. Cells were seeded at 20 and 40 million cells/mL in the case of A-172 and U-251 MG, respectively. The final collagen hydrogel solution was prepared at 2 mg/mL. Chips were placed inside the incubator (37 °C, 5% CO_2_) for 24 or 48 h to achieve a central hypoxic zone. Then, NNC was added at a final concentration of 10 µM through the lateral channels and left for 24 h (*n* = 5 for U-251 MG and *n* = 4 for A-172). The same tests and culture conditions were performed on the control devices without adding NNC to the medium (*n* = 6 for U-251 MG and *n* = 4 for A-172). To observe the percentage of live/dead cells within the central chamber, calcein-AM/propidium iodide (CAM/PI) staining was performed. Stock solutions of 1 mg/mL CAM (Life Technologies C1430) and 2 mg/mL PI (Sigma-Aldrich P4170) were dissolved in DMSO and distilled water. CAM and PI stock solutions were diluted to 2 and 6 μg/mL, respectively, in phosphate-buffered saline (PBS; Lonza BE17-516F). Bottom layer was removed from the device, to release the hydrogel, and CAM/PI solution was added directly above the gel, ensuring that the entire hydrogel surface is in contact with the dye. Then, the solution was incubated for 20 min.

### Cell treatments and Western blot

About 250,000 cells were seeded on a p35 dish. Cells to be exposed to hypoxia were shifted to Whitley H35 Hypoxystation on the day after plating plates and kept under a humid atmosphere for 48 h at 1% O_2_, while parallel normoxic cultures remained in the usual tissue culture incubator. NNC 10 µM was added for the last 8 h. At the end of the treatment, cells were washed with phosphate buffer saline (PBS) and lysed with a solution containing Tris 62.5 mM, pH = 6.8 and 2% sodium dodecyl sulfate (SDS). Cell lysates were separated by SDS-polyacrylamide gel electrophoresis and transferred to a polyvinylidene difluoride (PVDF) membrane (Merck Millipore, Massachusetts, USA; IPVH00010). Membranes were cut to probe different antibodies on the same membrane. Membranes were blocked with 5% milk and incubated overnight with primary antibodies. Blots were developed using Enhanced Chemiluminescence (ECL Western Blotting Substrate, Thermo Fisher Scientific, Massachusetts, USA; 32106) or Immobilon Forte Western Horse Radish Peroxidase substrate (Merck-Millipore, Massachusetts, USA; WBLUF0100). Band intensity was measured using ImageJ software and normalised against β-actin band intensity. For Fig. 3, *n* = 6 for U-251 MG and *n* = 5 for A-172. For Fig. 4, *n* = 6–8, except for the bafilomycin positive control (*n* = 4–5).

### Immunofluorescence

Cells (175/mm^2^) were plated on 25 μg/mL poly-D-lysine (PDL)-coated glass coverslips and treated with 10 μM NNC 55-0396 or 50 nM bafilomicyn-A1 for 6–8 h. Cells were fixed with 4% paraformaldehyde for 15 min at room temperature. For microdevices, gels were fixed with 4% PFA for 45 min, and extracted from the microdevice (*n* = 3 for each condition). Then, cells were washed with PBS twice and permeabilised and blocked using PBS containing 5% FBS, 5% horse serum (Fisher Scientific, Massachusetts, USA; 26050088), 0.2% glycine, 0.1% Triton X-100 (Merck Sigma-Aldrich, Massachusetts, USA; T8787). Cells were incubated with primary antibodies overnight at 4 °C, washed and incubated secondary antibodies together with Hoechst 33258 (Merck Sigma-Aldrich, Massachusetts, USA; 861405). Coverslips were mounted on Mowiol (Mowiol4-88, glycerol 50%, 0.2 M Tris-HCl, pH 8.5). Images were obtained using an inverted Olympus IX70 microscope equipped with epifluorescence optics and camera (Olympus OM-4 Ti) and, in the case of microdevices, Nikon Eclipse Ti-E coupled to a C1 modular confocal microscope. DPM Manager and Fiji ImageJ Softwares were used to process the pictures.

### Statistical analyses, Image J and bioinformatics

Statistical significance was assessed by Student’s t-test and asterisks represent different significance levels in t-test (*p < 0.05; **p < 0.01; and ***p < 0.001). Additionally, two-way ANOVA was used and significance levels were indicated as: #*p* < 0.05; ##*p* < 0.01; ###*p* < 0.001. For the 3D model, normal distribution was tested by the Kolmogorov-Smirnov test, and two-way ANOVA was performed with the Holm-Sidak method for statistical significance. Data is represented as mean ± SEM (*n* values are indicated in each figure legend). HIF-1α mRNA expression levels were analysed using the Gliovis platform accessing two different datasets (TCGA and Rembrandt) and comparing between histological types of gliomas and GBM subtypes [48]. Survival curves depending on HIF-1α levels were obtained from Gliovis.

## Results

### NNC reduces HIF-1α levels in GBM cells under hypoxic conditions

**Fig. 2:**
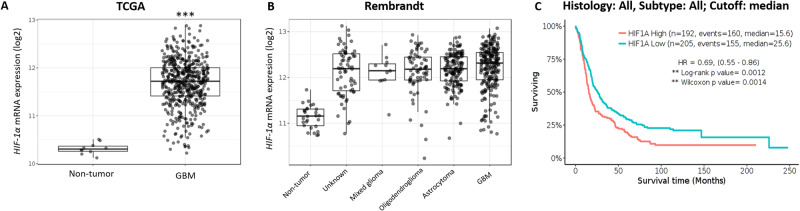
HIF-1α is overexpressed in GBM and confers worse prognosis. **A** HIF-1α is overexpressed in GBM (528 samples; TCGA dataset) compared to non-tumour samples (10 samples; mean 10.32 + 0.12 vs. 11.69 + 0.49; Paired t test (Tukey’s), *p* = 7.e−18). **B** Higher expression of HIF-1α in different glioma types, including GBM, compared to non-tumour (Rembrandt dataset; *p* < 0.001). **C** HIF-1α higher levels decrease patient survival (all gliomas; Rembrandt dataset).

The hypoxic/peri-necrotic niche drives adaptation to hypoxic conditions of solid tumours including GBM and is mainly directed by HIF-1*α* expression. We searched the TCGA and Rembrandt glioma datasets using the Gliovis platform and found that HIF-1α is overexpressed in GBMs compared to non-tumoral tissue (Fig. 2A, B) [48]. Importantly, HIF-1α levels correlated with prognosis, as glioma patients expressing higher levels of HIF-1α showed a median survival 10 months shorter than patients expressing lower levels of HIF-1α (15.6 vs 25.6 months, respectively; Fig. 2C). Consequently, modulating HIF-1α may be therapeutic in GBM.

**Fig. 3:**
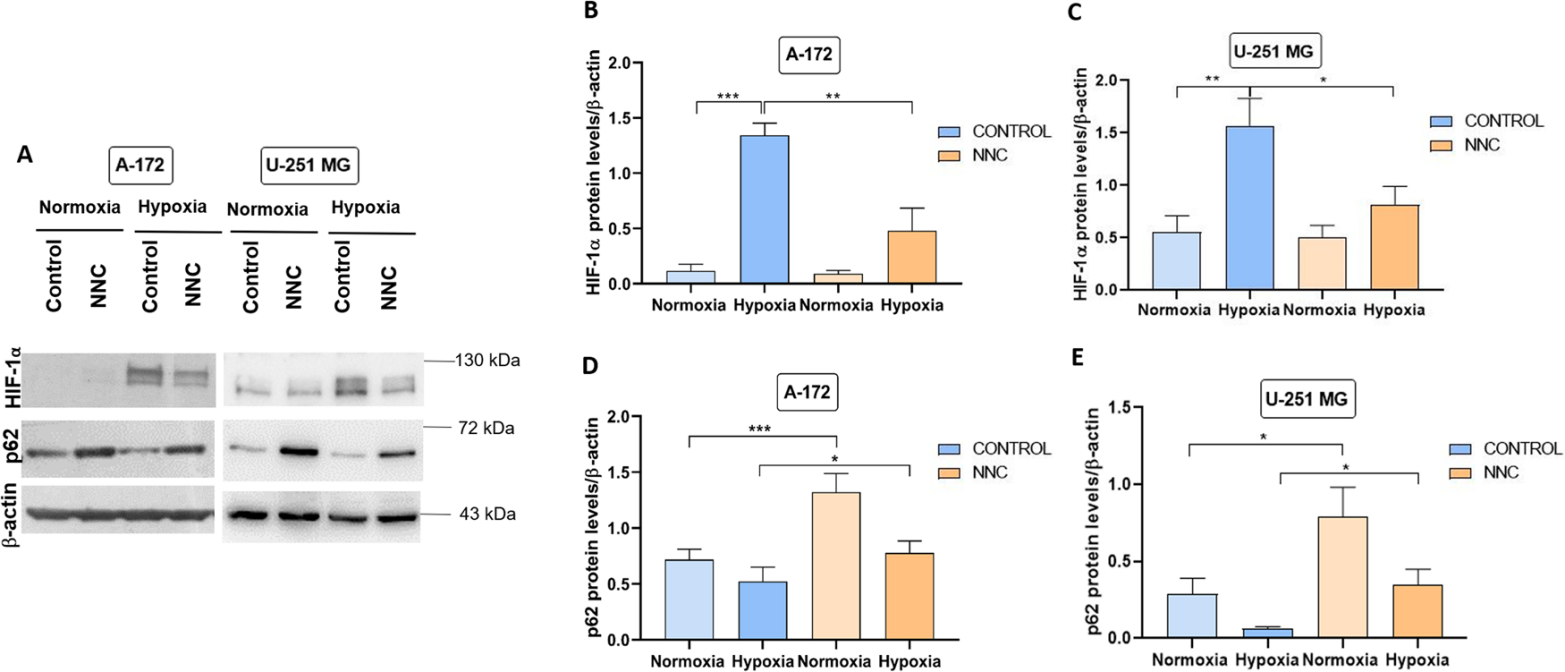
NNC reduces HIF-1α levels, while increasing p62. **A** Western-blot of HIF-1α and p62/SQSTM1 from A-172 and U-251 MG cells grown in normoxia or hypoxia for 48 h (untreated or treated with NNC for the last 8 h). β-actin was used as a loading control. **B**–**E** Quantification of HIF-1α (**B**, **C**) or p62 (**D**, **E**) levels (*p < 0.05; **p < 0.01; ***p < 0.005; *n* = 6 U-251 MG, *n* = 5 A-172). ANOVA analysis significance were # (panel **D**), ## (panel **C**) and ### (panels **B** and **E**).

We first analysed the effects of NNC (10 µM, 8 h) on HIF-1α levels using GBM cells (A-172 and U-251 MG GBM cell lines) grown under normoxia (20% O_2_) or hypoxia (1% O_2_) for 48 h. Cell lysates of NNC-treated samples from both cell lines showed a significant decrease in HIF-1α levels in hypoxia under the effect of NNC (Fig. 3A–C). Nonetheless, U-251MG cells showed basal detectable HIF-1α expression in normoxia, which similarly decreased by the effect of the drug.

### NNC blocks autophagy in GBM

**Fig. 4:**
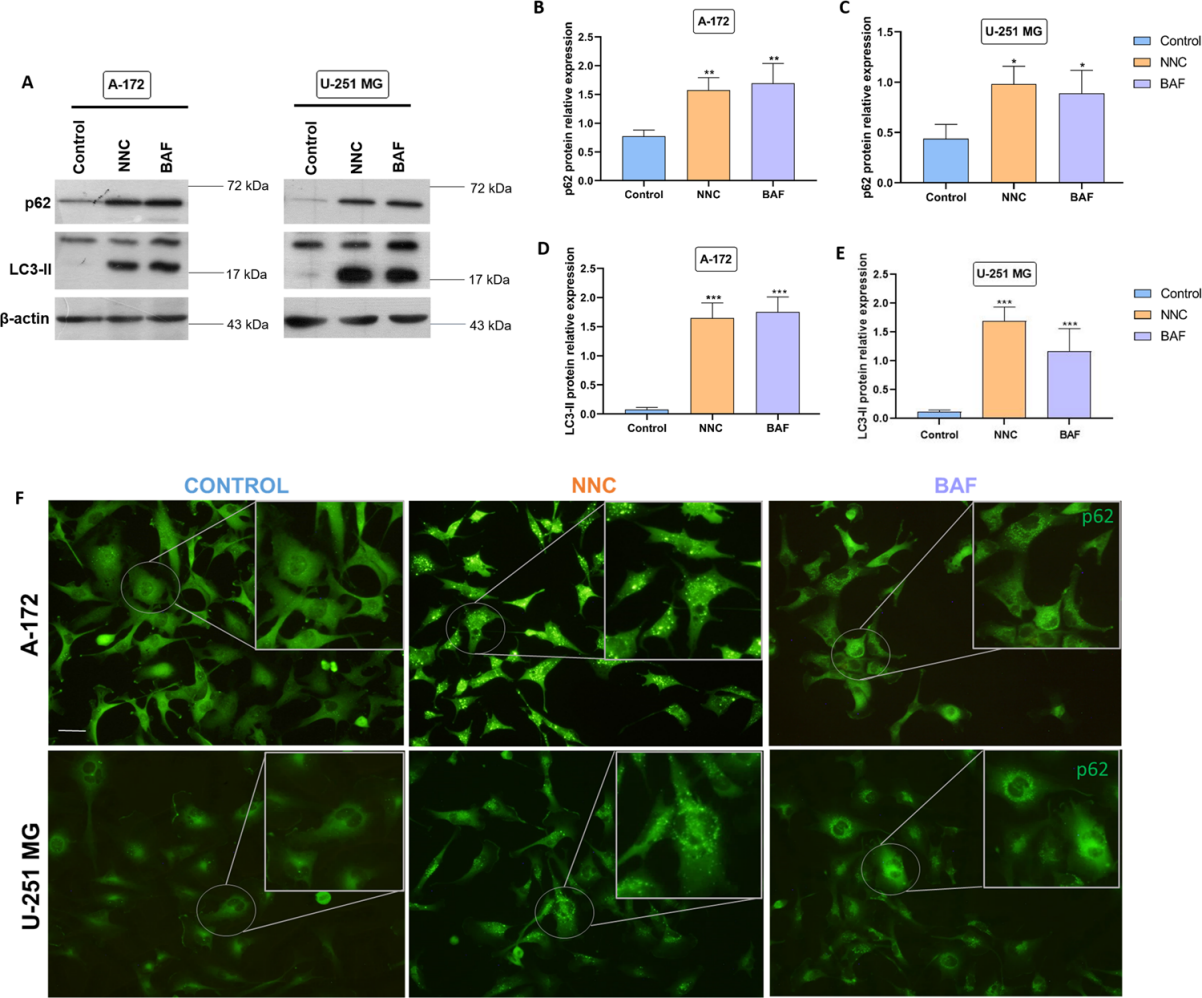
NNC blocks autophagy. **A** Western-blot of p62 and LC3-II from A-172 and U-251 MG cell lines untreated or treated with NNC or Baf (8 h). β-actin was used as a loading control. **B**, **C** Quantification of p62 levels. **D**, **E** Quantification of LC3-II levels (*p < 0.05; **p < 0.01; ***p < 0.005; *n* = 6–8 for NNC, *n* = 4–5 for Baf). **F** Immunostaining against p62 in both cell lines, untreated or treated as indicated. Note the dotted pattern with big puncta in NNC-treated cells. Scale bar: 40 µm.

In order to investigate if NNC affected macroautophagy, p62/SQSTM1, a cargo receptor of autophagosomes, was examined. Accordingly, we observed that NNC promoted a consistent increase of p62 protein levels (Fig. 3A, D, E). In addition, we analysed LC3-II, the lipidated form of the autophagosome protein LC3, to understand changes in autophagic flux induced by NNC. Then, we compared the effects of NNC and bafilomycin-C (Baf; 50 nM, 8 h), that inhibits the lysosomal v-ATPase and blocks autophagic flux. We found that NNC and Baf induced a similar accumulation of LC3-II and p62, compared to untreated control in both U-251 MG and A-172 cell lines (Fig. 4A–E). Furthermore, immunostaining experiments confirmed that NNC induced a clear increase in the number and size of p62 puncta compared to control cells, while Baf-treated cells displayed abundant fine p62 puncta (Fig. 4F). Altogether, these results indicate that NNC blocks macroautophagy at a late stage.

### NNC treatment induces cell death in the hypoxic areas of microfluidic devices

Microfluidic devices allowed us to investigate the response of GBM cells to NNC in a more physiologically relevant setting, mimicking the in vivo tumour microenvironment. Thanks to the 3D organisation, a complex tumour microenvironment was recreated, characterised by a hypoxic zone in the centre of the device, a starved zone in between and a zone with high viability due to high nutrient availability at the periphery (Fig. 1).

**Fig. 5:**
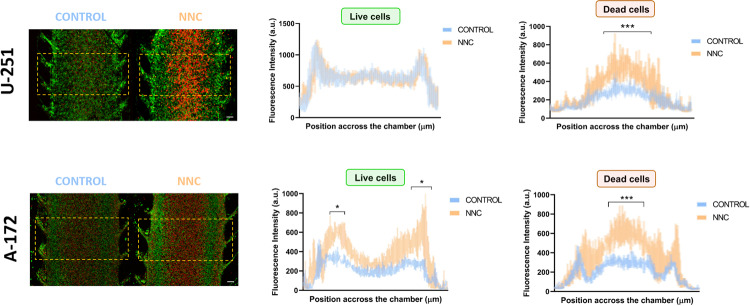
Effect of NNC application on GBM cells U-251 MG and A-172 in a GBM-on-a-chip model. On the left, confocal microscopy images of control devices and NNC treatment. Viability staining shows in green alive cells and in red dead cells. On the right, the graphs show the quantification of cellular fluorescence of alive and death cells in control (blue) and NNC-treated devices (orange). *p < 0.05; ***p < 0.001 denotes significant differences between conditions. *n* = 4–6 for each condition. The x-axis of the graphs shows the width used to analyse fluorescence, ranging from 0 to 2387 µm. Scale bar: 200 µm.

After 48 h, the model generated sufficient hypoxic conditions in the centre of the chamber to induce the formation of the central necrotic core observed in GBM, as demonstrated in Ayuso et al. [21]. At this moment, NNC was added and, 24 h later, the viability was assessed by confocal microscopy (Fig. 5). Consistent with the 2D experiments, treatment with NNC resulted in a significant increase (***p < 0.001) in cell death in the central hypoxic zone of both GBM cell lines (“Dead cells” Fig. 5). This effect was not observed in the control samples, indicating a pronounced response to the drug under hypoxic conditions. Conversely, cell death in the peripheral normoxic zones was not significantly affected by NNC treatment, further supporting the specificity of the drug’s action under restricted oxygen conditions. NNC effects were less evident in microdevices treated 24 h after the cell culture (Suppl. Figure 1), in which the hypoxic core was not fully established.

As expected, cell viability remained higher at the edges of the chip, particularly in the pillar area, both in the control and after drug application. This is likely due to the abundance of nutrients in this region, which creates a more favourable environment for tumour cell growth. Additionally, cells in normoxia appeared to be less sensitive to NNC and were thus able to proliferate in these areas (Suppl. Figure 2). However, in the case of the A-172 cell line, which exhibited higher on-chip metabolic activity than the U-251 MG line (Suppl. Figure 3), cell viability in the control decreased, which caused saturation of the chip and the inability of nutrients to reach the cells.

### NNC treatment blocks autophagy, especially in the normoxic regions of microfluidic devices

To investigate the causes underlying GBM cell viability reduction by NNC, we examined the autophagic flux in the aforementioned cell lines by immunofluorescent staining of p62 protein. These experiments aimed to confirm what had been observed in 2D, as well as to understand the spatial localisation of this marker within a 3D tumour in vitro.

**Fig. 6:**
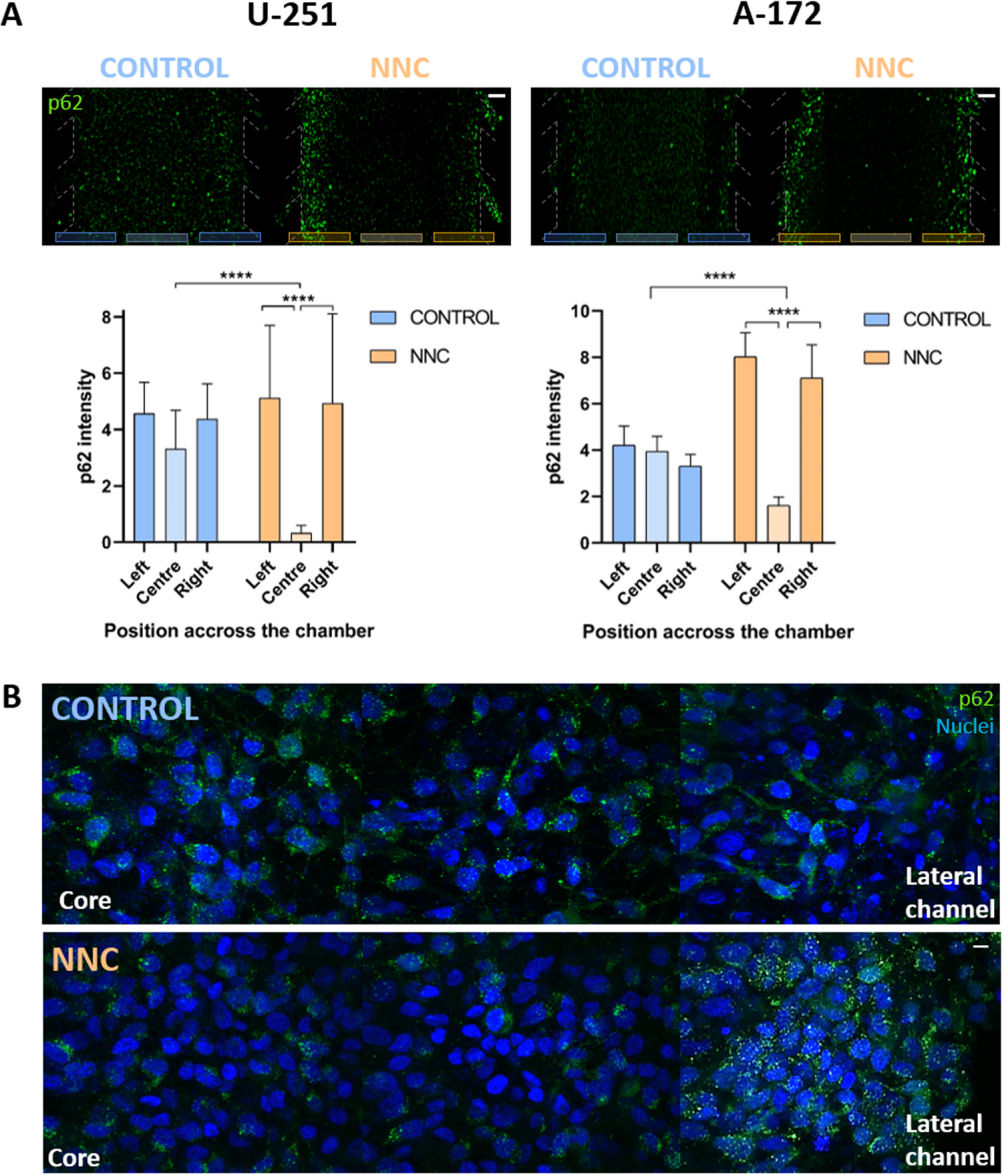
NNC treatment alters autophagy flux on GBM-on-a-chip model. **A** p62 immunofluorescence in microfluidic devices seeded with U-251 MG and A-172. Scale bar: 200 µm. The graphs below show the normalised levels of p62 fluorescence intensity in the left and right-side zones, and the central zone for both cell lines. **B** Images under confocal microscopy at 60x of p62 expression in U-251 MG. The left side corresponds to the centre of the chip (“Core”), where there is central hypoxia, while the right side corresponds to the region in contact with the side channels (“Lateral channel”). ***p < 0.001, *n* = 3 for each condition. Scale bar: 10 µm.

Our results showed that NNC treatment led to a significant accumulation and increase in p62 protein levels in the peripheral regions of the chip, *i.e*. in the normoxic region of the tumour (Fig. 6). The graphs in Fig. 6A illustrated a significant increase of p62 in the normoxic areas of NNC devices in both cell lines (***p < 0.001), compared to the control. It could be seen that the presence of p62 in the left and right peripheral areas increased by 195% and 215%, respectively, when NNC treatment was applied to the A-172 cell line. Similar data was obtained in the U-251 MG line. In this case, the lateral zones of the device also showed higher p62 levels, with 120% and 115% higher mean intensity in the left and right zones, respectively. Conversely, in the hypoxic zone of the NNC-treated chips of both cell lines, there seemed to be no accumulation of p62 protein, which could indicate a lower effect of the drug in blocking autophagic flux under hypoxia. However, as showed in Fig. 5, the drug induced greater cell death in the hypoxic zone in comparison to the normoxic area, where cells remained proliferative (Suppl. Figure 2) and the drug’s effect on p62 was more clearly visible.

## Discussion

Glioblastoma is a malignant tumour with a very poor prognosis, in part due to the great complexity and heterogeneity of its microenvironment [40]. The GBM central hypoxic core is a key factor leading to an invasive phenotype in tumour cells, which is a major contributor to tumour chemoresistance and malignancy [49]. In particular, HIF-1α is the main factor activated under hypoxia, which promotes pathways related to tumour progression as cell survival, invasion, angiogenesis, immunosuppression and metabolic reprogramming [50]. In addition, hypoxia induces the activation of autophagy, which allows tumour cells to resist oxygen starvation stress [51, 52]. In our study, a meta-analysis using data from 24 different studies showed that HIF-1α is overexpressed in GBM with respect to healthy tissue, and that patients with higher HIF-1α expression have shorter median survival. Besides, HIF-1α correlates with a higher tumour grade and have been pinpointed as a driver from low-grade gliomas to GBM [50, 53]. Furthermore, hypoxia has been shown to modulate the expression of TTCCs [54], which regulate cell cycle progression and chemoresistance in GBM [55]. Therefore, pharmacological targeting of TTCCs may be a therapeutic option to prevent GBM cells from adapting to hypoxia, thereby reducing tumour growth and resistance. In this context, the compound NNC-55-0396 has been described as a calcium channel blocker, although there is ample evidence indicating multiple other actions. Previous studies have shown that NNC at 10 µM promotes massive cell death of GBM cultures, through ER stress, overactivation of the Unfolded Protein Response (UPR) and calcium mobilisation [11, 55]. Here, we established a GBM-on-chip model to mimic the tumour microenvironment in a setting that closely emulates in vivo conditions [21]. This has allowed us to recreate different regions of the tumour; in particular, the hypoxic core typical of GBM, where to test the effects of NNC.

Lack of stabilisation of HIF-1α has been linked to reduced adaptation of tumour cells to hypoxic conditions [49]. In this respect, we found that NNC significantly reduces HIF-1α levels under hypoxic conditions in vitro in two GBM cell lines with different phenotypic characteristics (A-172 and U-251 MG). This result is consistent with Kim et al., in which NNC treatment destabilised HIF-1α in acute hypoxia (4 h), resulting in reduced tumorigenesis [10]. Nevertheless, U-251 MG cells show basal HIF1α levels in normoxia. Non-canonical mechanisms for HIF1α regulation have been described [56], and would explain these findings in U-251 MG cells. They include low activity of the Krebs cycle (associated with high glycolysis), with metabolites such as succinate and fumarate blocking PDH hydroxylase responsible of HIF1α degradation. Interestingly, Lu et al. [57] reported that inactivation of HIF-1α hydroxylation by glucose-derived 2-oxoacids underlies the basal HIF-1α activity common to highly glycolytic cancer cells like U-251 MG. In line with in vitro data, microfluidic devices seeded with GBM cells showed that NNC induces significant cell death in the central hypoxic zone of the tumour. Therefore, the drug preferentially targets this zone of the tumour, which has been described as one of the inducers of GBM malignancy and aggressiveness [58]. Currently, several HIF inhibitors are already showing promising results in preclinical studies. For example, cardiac glycosides have been used as HIF-1α inhibitors, reducing proliferation and inducing autophagy and/or apoptosis in cancer cells [59]; or LBH589, a panobinostat that also reduces HIF-1α and VEGF levels [60]. However, the lack of selectivity remains a major limitation in the validation of these drugs. Interestingly, lowering HIF-1α levels seems to be related to an increased sensibility to TMZ [61], suggesting that a chemotherapy treatment combining TMZ and NNC could be beneficial for GBM patients.

Additionally, data also shows a blockade of autophagy by NNC. Autophagy is a catabolic mechanism that occurs in response to nutrient starvation or oxidative stress, and ensures cell homoeostasis and survival [62]. In advanced tumour stages, this mechanism may promote the adaptation of GBM cells to nutrient starvation and hypoxia [7]. Western-blot and immunofluorescence assays show a significant accumulation of p62/SQSTM1 and LC3-II proteins in the presence of NNC compared to the untreated control in both A-172 and U-251 MG. These results suggest a blockade of the macroautophagy process at a late stage by NNC, as p62/SQSTM1 is degraded upon autophagosome-lysosome fusion. Accumulation of p62 is reduced in hypoxia compared to normoxia, which is consistent with its increased degradation in hypoxia [63]. By performing immunofluorescence in our GBM-on-a-Chip model, we could observe a clear accumulation of p62 in the outermost areas of the tumour after NNC application compared to untreated chips from both GBM cell lines. This shows a blockade of autophagy by NNC in the periphery of the tumour, which makes up the potentially invasive zone of the tumour. Autophagy blockade could be governing the destabilisation of HIF-1α and cell death in hypoxic areas. Kim et al. reported that proteasome inhibitor MG132 was able to revert the NNC-decreased HIF-1α levels [10]. Thus, a potential overactivation of the proteasome by NNC may be consequence of the defective autophagy [64].

Altogether, we conclude that NNC predominantly induces cell death in the hypoxic central zone of the GBM by reducing HIF-1α. In addition, the drug blocks adaptive autophagy in the most active regions of the tumour. Both actions contribute to previously described effects of the drug in cancer cells, that therefore co-targets UPR, autophagy and hypoxia adaptation, in which the organ-on-chip model provides greater biomimicry and spatial information. Thus, NNC administration could be a potential treatment for GBM, designed to deplete the hypoxic tumour region and to reduce treatment resistance of this aggressive brain cancer.

### Supplementary information

Supplementary figures legend

Supplementary figure 1

Supplementary figure 2

Supplementary figure 3

Original Data File

checklist

## Data Availability

Additional data can be found in Supplementary information.
