# Supplementary material for: Tetralol derivative NNC-55-0396 targets hypoxic cells in the glioblastoma microenvironment: an organ-on-chip approach
Source: Cell Death Dis. 2024 Feb 10;15(2):127. doi: 10.1038/s41419-024-06492-1 (PMC10858941; doi:10.1038/s41419-024-06492-1)
Supplement: Supplementary file 1 — Supplementary figures legend [file 41419_2024_6492_MOESM1_ESM.docx]

**Supplementary information**

**Supple. Fig 1**. NNC effect in devices with only 24 hours of core formation. The lack of a fully formed hypoxic core decreases the effect of the drug as there is less hypoxia. In both U-251 MG (top) and A-172 (bottom) cell lines, no significant differences in viability and cell death between the control and NNC-treated samples were observed. Scale bar: 200 µm.

**Supple. Fig 2**. Cell proliferation based on ki-67 expression in GBM on a chip. A proliferative phenotype of U-251 MG cells is observed in the areas adjacent to the side channels with the presence of nutrients and oxygen, while in the central area there is practically no expression of this proliferation marker. The Ki-67 expression is shown in green, and the nuclei in blue. Scale bar: 200 µm.

**Supple. Fig 3**. High metabolic activity of A-172 cells. Image of a microfluidic device seeded with 40 mill/mL A-172 cells after 48 hours of culture. It can be seen that when seeding these cells at the same density as U-251 MG, the central necrotic zone occupies practically most of the central chamber, and only a few viable cells remain in the region where nutrients and oxygen enter.
