# Supplementary figures and images for: Tetralol derivative NNC-55-0396 targets hypoxic cells in the glioblastoma microenvironment: an organ-on-chip approach
Source: Cell Death Dis. 2024 Feb 10;15(2):127. doi: 10.1038/s41419-024-06492-1 (PMC10858941; doi:10.1038/s41419-024-06492-1)

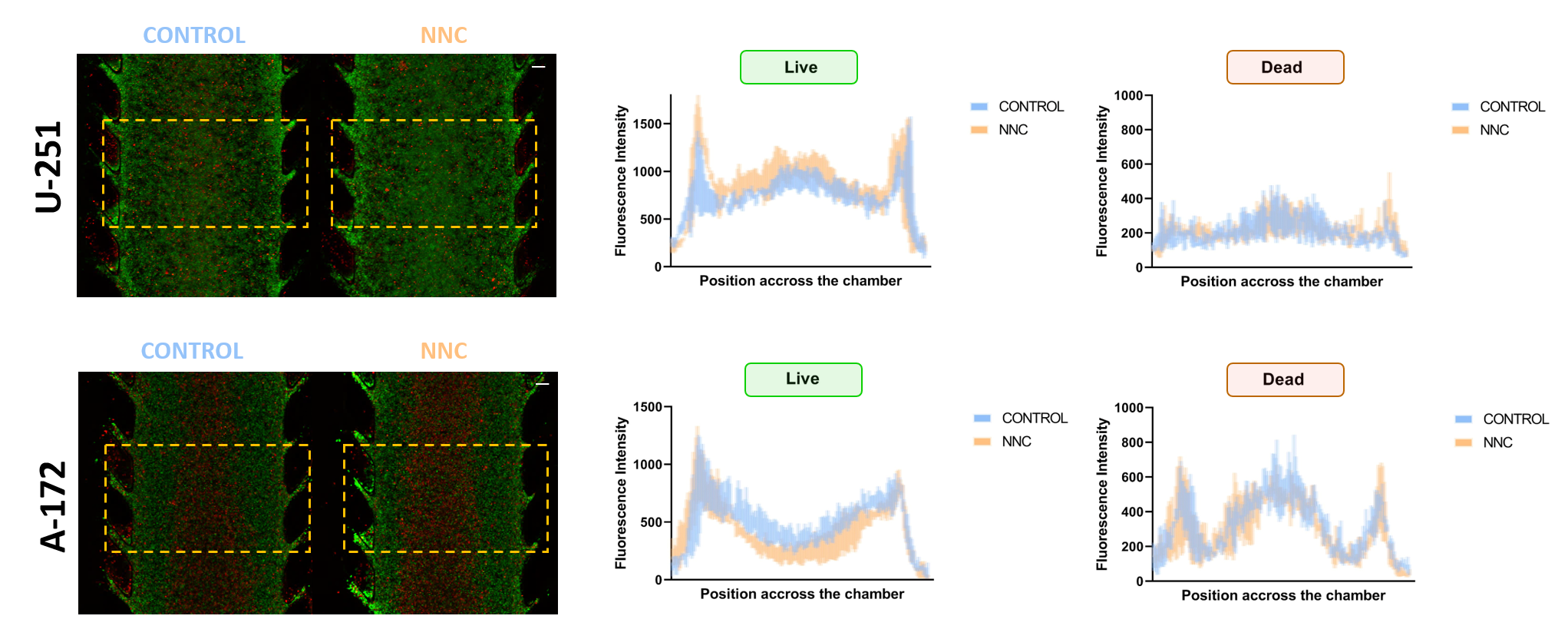

Supplement: Supplementary file 2 — Supplementary figure 1 [file 41419_2024_6492_MOESM2_ESM.png]

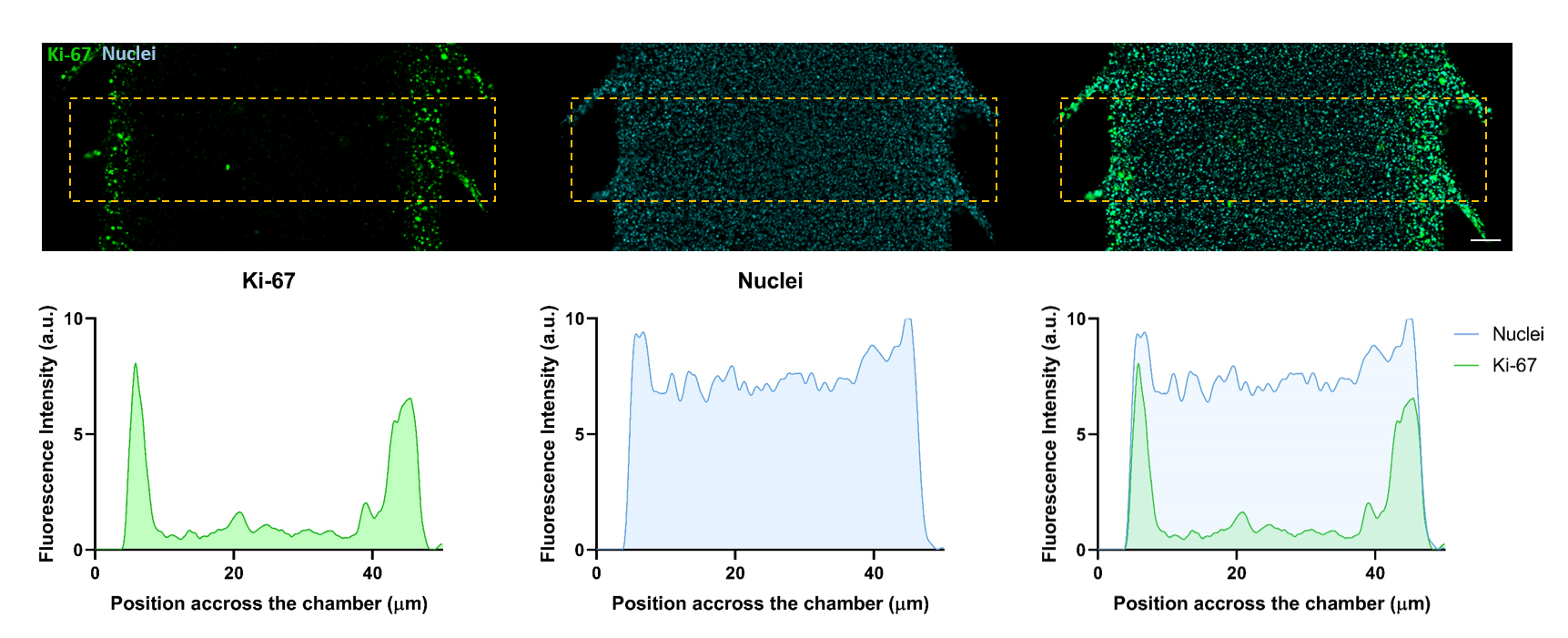

Supplement: Supplementary file 3 — Supplementary figure 2 [file 41419_2024_6492_MOESM3_ESM.png]

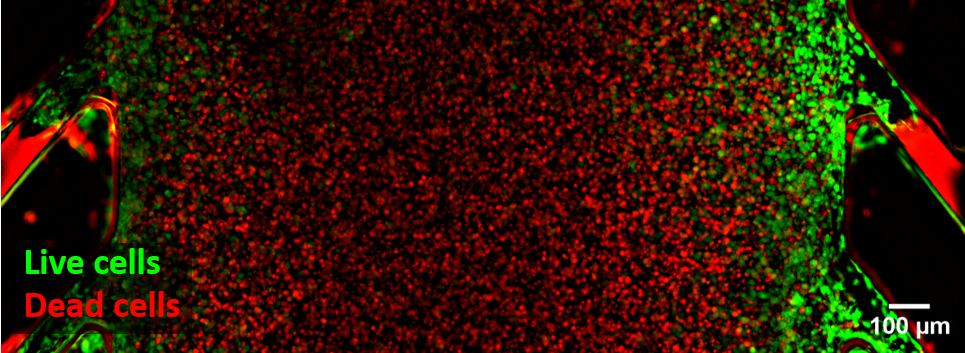

Supplement: Supplementary file 4 — Supplementary figure 3 [file 41419_2024_6492_MOESM4_ESM.png]

Fig 3A

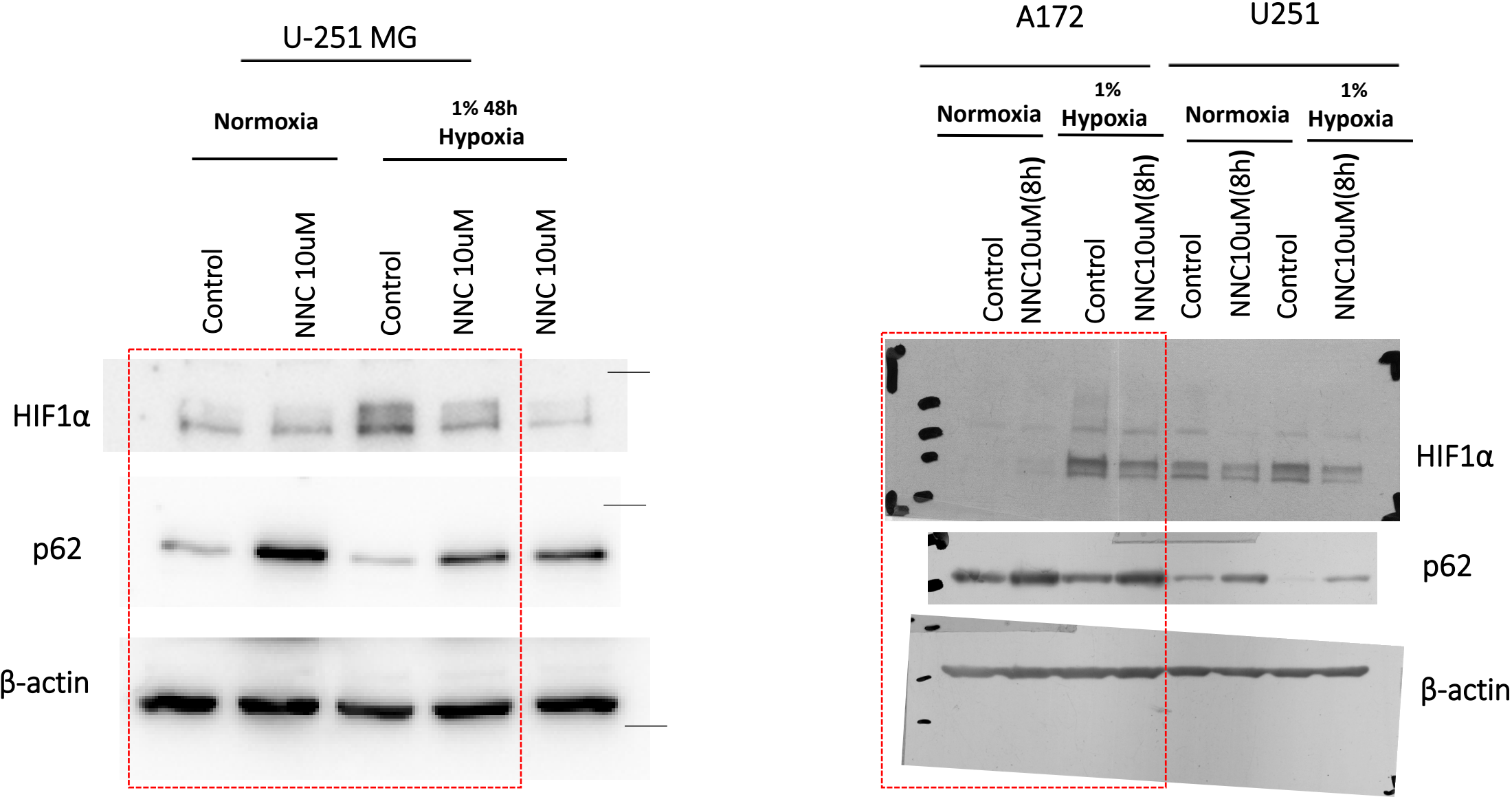

Fig 4A

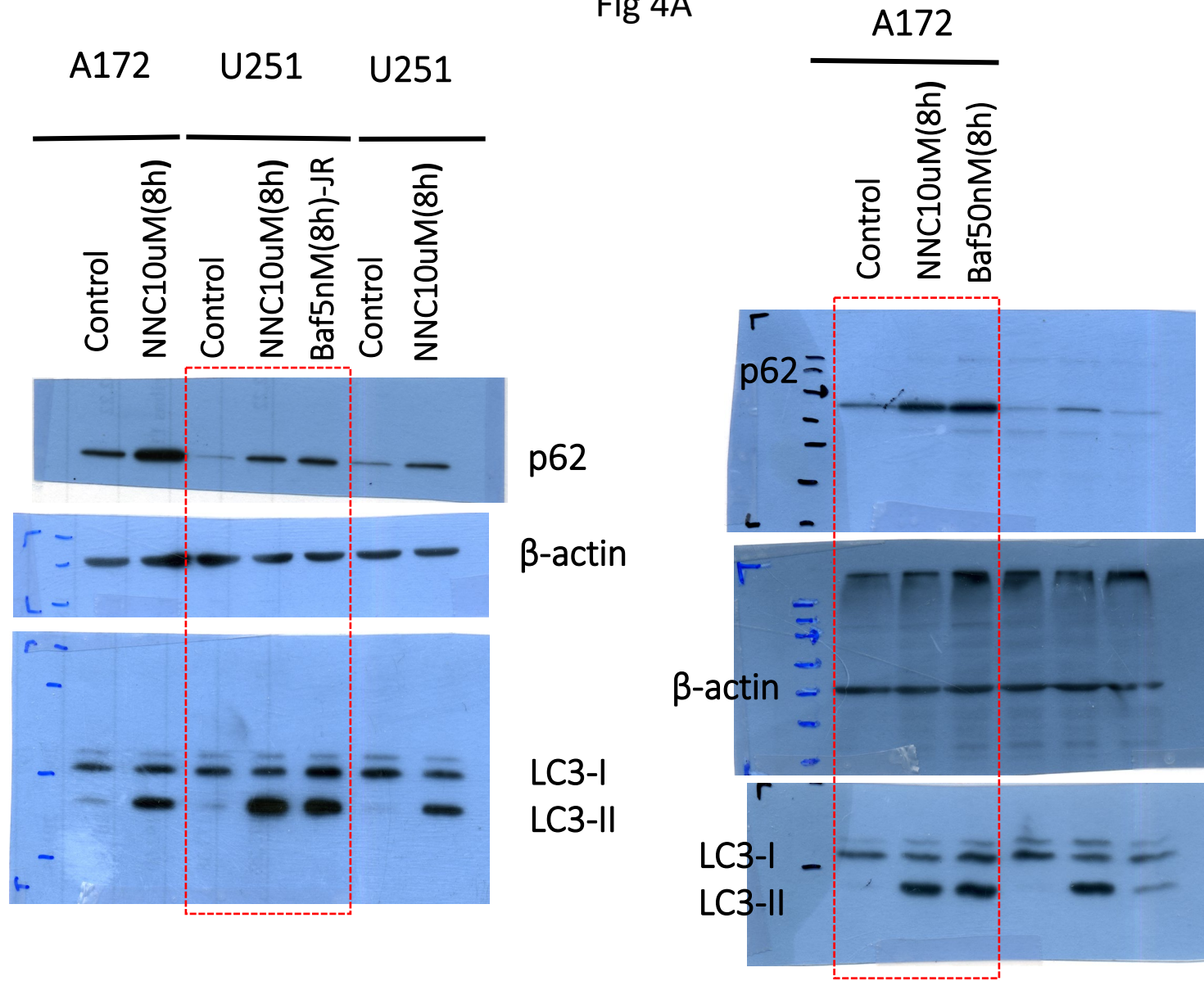

Supplement: Supplementary file 5 — Original Data File [file 41419_2024_6492_MOESM5_ESM.pdf]
